# Joint Analysis of Cortical Area and Thickness as a Replacement for the Analysis of the Volume of the Cerebral Cortex

**DOI:** 10.1093/cercor/bhx308

**Published:** 2017-11-28

**Authors:** Anderson M Winkler, Douglas N Greve, Knut J Bjuland, Thomas E Nichols, Mert R Sabuncu, Asta K Håberg, Jon Skranes, Lars M Rimol

**Affiliations:** 1 Oxford Centre for Functional MRI of the Brain, Nuffield Department of Clinical Neurosciences, University of Oxford, Oxford OX3 9DU, UK; 2 Department of Psychiatry, Yale University School of Medicine, New Haven, CT 06511, USA; 3 Big Data Analytics Group, Hospital Israelita Albert Einstein, São Paulo, SP 05652-900, Brazil; 4 Martinos Center for Biomedical Imaging, Massachusetts General Hospital/ Harvard Medical School, Charlestown, MA 02129, USA; 5 Department of Laboratory Medicine, Children’s and Women’s Health, Norwegian University of Science and Technology, Trondheim 7030, Norway; 6 Oxford Big Data Institute, Li Ka Shing Centre for Health Information and Discovery, Nuffield Department of Population Health, University of Oxford, Oxford OX3 7LF, UK; 7 School of Electrical and Computer Engineering, and Meinig School of Biomedical Engineering, Cornell University, Ithaca, NY 14853, USA; 8 Department of Neuroscience, Norwegian University of Science and Technology, Trondheim 7030, Norway; 9 Department of Radiology, St. Olav's Hospital, Trondheim University Hospital, Trondheim 7030, Norway; 10 Department of Pediatrics, Sørlandet Hospital, 4838 Arendal, Norway; 11 Norwegian Advisory Unit for Functional MRI, Department of Radiology, St. Olav’s University Hospital, Trondheim 7006, Norway

**Keywords:** cortical gray matter volume, cortical surface area, cortical thickness, nonparametric combination

## Abstract

Cortical surface area is an increasingly used brain morphology metric that is ontogenetically and phylogenetically distinct from cortical thickness and offers a separate index of neurodevelopment and disease. However, the various existing methods for assessment of cortical surface area from magnetic resonance images have never been systematically compared. We show that the surface area method implemented in FreeSurfer corresponds closely to the exact, but computationally more demanding, mass-conservative (pycnophylactic) method, provided that images are smoothed. Thus, the data produced by this method can be interpreted as estimates of cortical surface area, as opposed to areal expansion. In addition, focusing on the joint analysis of thickness and area, we compare an improved, analytic method for measuring cortical volume to a permutation-based nonparametric combination (NPC) method. We use the methods to analyze area, thickness and volume in young adults born preterm with very low birth weight, and show that NPC analysis is a more sensitive option for studying joint effects on area and thickness, giving equal weight to variation in both of these 2 morphological features.

## Introduction

It has been suggested that biological processes that drive horizontal (tangential) and vertical (radial) development of the cerebral cortex are separate from each other (Rakic 1988; Geschwind and Rakic 2013), influencing cortical area and thickness independently. These 2 indices of cerebral morphology are uncorrelated genetically (Panizzon et al. 2009; Winkler et al. 2010), are each influenced by regionally distinct genetic factors (Schmitt et al. 2008; Rimol, Panizzon, et al. 2010; Chen et al. 2012, 2015), follow different trajectories over the lifespan (O’Leary et al. 2007; Hogstrom et al. 2013; Fjell et al. 2015), and are differentially associated with cognitive abilities and disorders (Noble et al. 2015; Schnack et al. 2015; Lee et al. 2016; Vuoksimaa et al. 2016). Moreover, it is cortical area, not thickness, that differs substantially across species (Rakic 1995). These findings give prominence to the use of surface area in studies of cortical morphology and its relationship to function. However, a number of approaches and terminologies exist for its assessment, which have not been studied in detail or compared directly, making interpretation and comparison between studies challenging. A first objective of this article is to compare the methods for the analysis of cortical area, in particular interpolation between surfaces at different resolutions, and to provide recommendations for users.

A second objective of the article is to demonstrate that a statistical joint analysis of cortical thickness and surface area, using the recently proposed nonparametric combination (NPC) (Pesarin and Salmaso 2010a; Winkler, Webster, et al. 2016), can be used to investigate factors affecting cortical morphology. While analyzing cortical thickness and cortical area separately improves specificity over combined metrics such as cortical volume (Rimol et al. 2012), it may still be of interest to jointly analyze these 2 measurements so as to increase power to detect effects on thickness and area simultaneously. In principle, this could be accomplished through the analysis of cortical volume, which commingles thickness and area. Indeed, volume is a popular metric, thanks mainly to the wide use of voxel-based morphometry (VBM) (Ashburner and Friston 2000; Good et al. 2001; Douaud et al. 2007), despite a series of well documented disadvantages (Davatzikos 2004; Ashburner 2009). In surface-based approaches, cortical volume is measured as the product of cortical thickness and surface area at each location across the cortical mantle. However, here we demonstrate that this multiplicative method incurs severe bias, the direction of which varies according to the local geometry of the cortex. In order to conduct a fair comparison of surface-based cortical volume analysis and joint analysis with NPC, we propose a novel, geometrically exact, analytic solution to the measurement of cortical volume, which does not suffer from such bias, and compare this improved cortical volume method to analysis with NPC.

### Cortical Surface Area

Using continuous (vertexwise) cortical maps to compare surface area across subjects has the advantage that, unlike approaches based on regions of interest (ROI), it does not depend on the effects of interest mapping onto a previously defined ROI scheme. However, few studies using continuous maps have offered insight into the procedures adopted. Sometimes the methods were described in terms of areal expansion/contraction, as opposed to surface area itself, and different definitions of areal expansion/contraction have been used, for example, relative to the contra-lateral hemisphere (Lyttelton et al. 2009), to some earlier point in time (Hill et al. 2010), to a control group (Palaniyappan et al. 2011), or in relation to a standard brain, possibly the default brain (average or atlas) used in the respective software package (Joyner et al. 2009; Rimol, Agartz, et al. 2010; Chen et al. 2011, 2012; Rimol et al. 2012; Vuoksimaa et al. 2016); other studies considered linear distances as proxies for expansion/contraction (Sun, Phillips, et al. 2009; Sun, Stuart, et al. 2009). Some of the studies that used a default brain as reference used nearest neighbor interpolation followed by smoothing, which, as we show below, assesses cortical area itself, but described the measurements in terms of areal expansion (Joyner et al. 2009; Rimol, Agartz, et al. 2010; Rimol et al. 2012).

Surface area analyses depend on registration of the cortical surface and interpolation to a common resolution; such resampling must preserve the amount of area at local, regional and global scales, that is, it must be mass-conservative. This means that the choice of interpolation method is crucial. A well-known interpolation method is nearest-neighbor, which can be enhanced by correction for stretches and shrinkages of the surface during registration, as available in the function mris_preproc, part of the FreeSurfer (FS) software package (Available at freesurfer.net). Another approach is retessellation of the mesh of the individual subject to the geometry of a common grid, as proposed by Saad et al. (2004) as a way to produce meshes with similar geometry across subjects. Even though the method has been mostly used to compute areal expansion, it can be used for surface area itself, as well as for other areal quantities. A third approach is the use of the barycentric coordinates of each vertex with reference to the vertices of the common grid to redistribute the areal quantities, in an approximately mass-conservative process. Lastly, a strategy for analysis of areal quantities using a pycnophylactic (mass-preserving) interpolation method, which addresses the above concerns but is computationally intensive, was presented in Winkler et al. (2012) (Table 1).

**Table 1:** Overview of the 4 different methods to interpolate surface area and areal quantities. A detailed description is in the Materials and Methods

Method	Description
Nearest neighbor	Nearest neighbor interpolation of areal quantities on the sphere, followed by Jacobian correction.
Retessellation	Barycentric interpolation on the sphere of the native vertex coordinates.
Redistributive	Vertexwise redistribution of areal quantities based on barycentric coordinates of the source in relation to the target.
Pycnophylactic	Mass-conservative facewise interpolation method that uses the overlapping areas between faces of source and target.

### Measuring Volume

**Figure 1.**
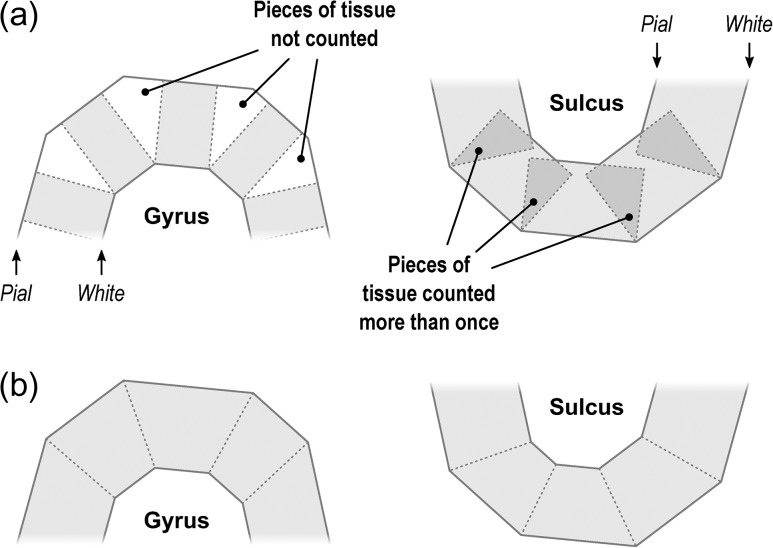
A diagram in 2 dimensions of the problem of measuring cortical volume. (*a*) If volume is computed using multiplication of thickness by area, considerable amount of tissue is left unmeasured in the gyri, or measured repeatedly in sulci. The problem is minimized, but not solved, with the use of the mid-surface. (*b*) Instead, vertex coordinates can be used to compute analytically the volume of tissue between matching faces of white and pial surfaces, leaving no tissue under- or over-represented.

**Figure 2.**
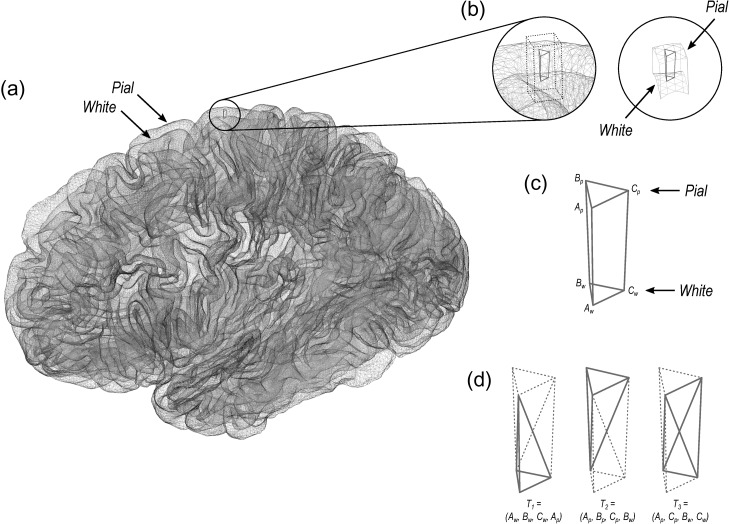
(*a*) In the surface representation, the cortex is limited internally by the white and externally by the pial surface. (*b*) and (*c*) These 2 surfaces have matching vertices that can be used to delineate an oblique truncated triangular pyramid. (*d*) The 6 vertices of this pyramid can be used to define 3 tetrahedra, the volumes of which can be computed analytically.

The volume of cortical gray matter is also an areal quantity, which therefore requires mass-conservative interpolation methods. Volume can be estimated through the use of voxelwise partial volume effects using a volume-based representation of the brain, such as in VBM, or from a surface representation, in which it can be measured as the amount of tissue present between the surface placed at the site of the pia mater, and the surface at the interface between gray and white matter. If the area of either of these surfaces is known, or if the area of a mid-surface, that is, the surface running half-distance between pial and white surfaces (Van Essen 2005) is known, an estimate of the volume can be obtained by multiplying, at each vertex, area by thickness. This procedure, while providing a reasonable approximation that improves over voxel-based measurements since it is less susceptible to various artefacts (for a discussion of artefacts in VBM, see Ashburner 2009), is still problematic as it underestimates the volume of tissue that is external to the convexity of the surface, and overestimates volume that is internal to it; both cases are undesirable, and cannot be solved by merely resorting to using an intermediate surface as the mid-surface (Fig. 1*a*). Here a different approach is proposed: each face of the white surface and its matching face in the pial surface are used to define an oblique truncated pyramid, the volume of which is computed analytically, without introducing additional error other than what is intrinsic to the placement and resolution of these surfaces (Fig. 1*b* for a 2D schema and Fig. 2 for a similar in 3D).

### Nonparametric Combination

We argue that analyzing thickness and area jointly offers important advantages over using cortical volume, regardless of how the latter is measured. The permutation-based NPC (Pesarin and Salmaso 2010a; Winkler, Webster, et al. 2016) supplies a test for directional as well as two-tailed hypotheses, which has been proven to be more powerful than classical multivariate tests (Pesarin and Salmaso 2010b). The NPC consists of, in a first phase, testing separately hypotheses on each available metric (here thickness and area) using permutations that are performed in synchrony; these tests are termed partial tests. The resulting statistics for every permutation are recorded, allowing an estimate of the complete empirical cumulative distribution function (cdf) to be constructed for each metric. In a second phase, the empirical *P*-values are combined, for each permutation, into a joint statistic. As the joint statistic is produced from the previous permutations that have been recorded, an estimate of its empirical cdf is immediately known, and therefore its *P*-value. The test is based on minimal assumptions, mainly that of exchangeability, that is, swapping one datum for another keeps the data just as likely. Independence among metrics or partial tests is not assumed by NPC: the synchronized permutations implicitly capture eventual dependencies. This is particularly important when investigating cortical area and thickness, since shared environmental effects may affect area and thickness simultaneously.

## Materials and Methods

We apply the methods to a cohort of adults born preterm with very low birth weight (≤1500 g, VLBW), and a set of coetaneous controls born at the same hospital and period. At age 20, from an initial group of 121 VLBW and 120 control subjects, a total of 41 VLBW and 59 controls consented to participate and had usable MRI data. Details about the sample can be found in Martinussen et al. (2005), Skranes et al. (2007). The local Regional Committee for Medical Research Ethics approved the study protocol (Norwegian Health Region IV; REK project number: 4.2005.2605). Previous studies of the preterm population have shown reduced cortical surface area in multiple frontal, temporal and parieto-occipital regions, as well as increased frontal lobe and reduced parietal lobe cortical thickness. This combination of reductions and increases, in partly overlapping regions, makes the present sample well-suited for a demonstration of joint analysis of surface area and cortical thickness. Using this data we evaluate the 4 different interpolation methods (nearest neighbor, retessellation, redistributive and pycnophylactic), how they interact with different resolutions (shown in the Supplementary Material only), the 2 ways of measuring volume (the product method and the analytic method) and, finally, we demonstrate benefits of NPC over cortical volume for the investigation of differences in cortical morphology between the 2 groups. We note that comparisons among interpolation methods depend only on algorithmic and geometric differences between them, not interacting with particular features of this or any sample, such that the results are generalizable and not influenced by pathology.

### Data Acquisition and Surface Reconstruction

MRI scanning was performed on a 1.5 T Siemens MAGNETOM Symphony scanner. Two sagittal T_1_-weighted magnetization prepared rapid gradient echo (MPRAGE) scans were acquired (TE/TR/TI = 3.45/2730/1000 ms, flip angle = 7 degrees, voxel size = 1 × 1 × 1.33 mm^3^). Surfaces were reconstructed using the FreeSurfer software package (version 5.3.0; Dale et al. 1999; Fischl, Sereno, and Dale 1999), and an overview of the whole process is in Supplementary Material S1. We used the FreeSurfer software suite but similar methods for surface reconstruction exist in other software packages (Mangin et al. 1995; Van Essen et al. 2001; Kim et al. 2005), and the present comparisons of interpolation methods and methods of volume measurement, as well as analysis with NPC, are not specific to FreeSurfer.

### Measurement of Areal Quantities

Areal quantities are measured in native space, that is, before registration. For the retessellation method, the measurement is made in native space after the surface has been reconstructed to a common grid; nearest neighbor, redistributive and pycnophylactic use native space measurements with the original, subject-specific mesh geometry.

#### Cortical Area

For a triangular face ABC of the surface representation, with vertex coordinates $a=[x_{A}y_{A}z_{A}]\prime$, $b=[x_{B}y_{B}z_{B}]\prime$ and $c=[x_{C}y_{C}z_{C}]\prime$, the area is |u×v|/2, where u=a−c, v=b−c, × represents the cross product, and the bars | | represent the vector norm. Although the area per face (i.e., the facewise area) can be used in subsequent steps, it remains the case that most software packages can only deal with values assigned to each vertex of the mesh (i.e., vertexwise). Conversion from facewise to vertexwise is achieved by assigning to each vertex one-third of the sum of the areas of all faces that have that vertex in common (Winkler et al. 2012).

#### Cortical Volume

The conventional method for computing surface-based volume consists of computing the area at each vertex as above, then multiplying this value by the thickness at that vertex, in a procedure that leaves tissue under- or over-represented in gyri and sulci (Fig. 1). We propose that, instead, volumes are computed using the 3 vertices that define a face in the white surface and the 3 matching vertices in the pial surface, defining an oblique truncated triangular pyramid, which in its turn is subdivided into 3 tetrahedra. The volumes of these are computed analytically, summed, and assigned to each face of the surface representation, that is:

1. For a given face $A_{w}B_{w}C_{w}$ in the white surface, and its corresponding face $A_{p}B_{p}C_{p}$ in the pial surface, define an oblique truncated triangular pyramid.
2. Split this truncated pyramid into 3 tetrahedra, defined as follows:
  $$\begin{array}{cc} T_{1} & =\;(A_{w},B_{w},C_{w},A_{p})\\ T_{2} & =\;(A_{p},B_{p},C_{p},B_{w})\\ T_{3} & =\;(A_{p},C_{p},B_{w},C_{w}) \end{array}$$
  This division leaves no volume under- or over-represented.
3. For each such tetrahedra, let a, b, c, and d represent its 4 vertices in terms of coordinates [x y z]′. Compute the volume as |u⋅(v×w)|/6, where u=a−d, v=b−d, w=c−d, the symbol × represents the cross product, ⋅ represents the dot product, and the bars | | represent the vector norm.

Computation can be accelerated by setting $d=A_{p}$, the common vertex for the 3 tetrahedra, such that the vector subtractions need to be performed only once. Conversion from facewise volume to vertexwise is possible, and done in the same manner as for facewise area. The above method for measuring volume has become the default in the current FreeSurfer version (6.0.0).

### Spherical Transformation and Registration

The white surface is homeomorphically transformed to a sphere (Fischl, Sereno, Tootell, et al. 1999), thus keeping a one-to-one mapping between vertices of the native geometry and the sphere. Various strategies are available to place these surfaces in register with a common reference and allow intersubject comparisons, including the method used by FreeSurfer (Fischl, Sereno, Tootell, et al. 1999), spherical demons (SD) (Yeo et al. 2010), multimodal surface matching (MSM) (Robinson et al. 2014), among others. Methods that are not diffeomorphic by design but in practice produce invertible and smooth warps can, in principle, be used in registration for areal analyses. In the present analyses, FreeSurfer was used (a complementary comparison with SD is shown in Supplementary Material S2). The measurements of interest obtained from native geometry or in native space, such as area and thickness, are stored separately and are not affected by the spherical transformation or registration.

### Interpolation Methods

Statistical comparisons require meshes with a common resolution where each point represents homologous locations across individuals. A geodesic sphere has many advantages for this purpose: ease of computation, edges of roughly similar sizes and, if the resolution is fine enough, edge lengths that are much smaller than the diameter of the sphere (Kenner 1976). We compared 4 different interpolation methods, described below, at each of 3 different mesh resolutions: IC3 (642 vertices and 1280 faces), IC5 (10 242 vertices and 20480 faces) and IC7 (163 842 vertices and 327 680 faces); for the comparison between VLBW and controls, the resolution used was IC7, with nearest neighbor interpolation.

#### Nearest Neighbor

The well-known nearest neighbor interpolation does not guarantee preservation of areal quantities, although modifications can be introduced to render it approximately mass conservative: for each vertex in the target, the closest vertex is found in the source sphere, and the area from the source vertex is assigned to the target vertex; if a given source vertex maps to multiple target vertices, its area is divided between them so as to preserve the total area. If there are any source vertices that have not been represented in the target, for each one of these, the closest target vertex is located and the corresponding area from the source surface is incremented to any area already stored on it. This method ensures that total area remains unchanged after mapping onto the group surface. This process is a surface equivalent of the Jacobian correction used in volume-based methods in that it accounts for stretches and shrinkages while preserving the overall amount of areal quantities. It should not be confused with the computation of the Jacobian itself, that is defined, for the i-th vertex, as $J_{i}=\frac{A_{i}^{S}}{A_{i}^{w}}\frac{\underset{j}{\sum }A_{j}^{w}}{\underset{j}{\sum }A_{j}^{S}}$, where $A_{i}^{S}$ is the area of the vertex in the source (registered) sphere, $A_{i}^{w}$ is the area of the same vertex in the white surface (native space and native geometry), and the sums are over the entire surface, i.e., all vertices. Nearest neighbor interpolation is the default method in FreeSurfer.

#### Retessellation of the Native Geometry

This method appeared in Saad et al. (2004). It consists of generating a new mesh by interpolating the coordinates of the vertices in the native geometry to the common grid, thus defining a new surface. The coordinates of each vertex can be treated as a single vector and barycentric interpolation performed in a single step, as follows:

$$\left[\begin{array}{c} x_{P}\\ y_{P}\\ z_{P} \end{array}\right]=\left[\begin{array}{ccc} x_{A} & x_{B} & x_{C}\\ y_{A} & y_{B} & y_{C}\\ z_{A} & z_{B} & z_{C} \end{array}\right]\left[\begin{array}{c} \delta_{A}\\ \delta_{B}\\ \delta_{C} \end{array}\right]$$

where x,y,z represent the coordinates of the triangular face ABC and of the interpolated point P, all in native geometry, and δ are the barycentric coordinates of P with respect to the same face after the spherical transformation. Among the 4 methods considered in this chapter, this is the only one that does not directly interpolate either area or areal quantities, but the mesh. The area for each face or vertex can then be computed from the new mesh and used for statistical analyses.

#### Redistribution of Areas

This method works by splitting the areal quantity present at each vertex in the source sphere using the proportion given by the barycentric coordinates of that vertex in relation to the face on which it lies in the target sphere, thus redistributing these quantities to the 3 vertices that constitute that face in the target. If some quantity was already present in the target vertex (e.g., from other source vertices lying on the same target face), that quantity is incremented. This method can be represented as follows:

$$Q_{i}^{T}=\overset{F}{\underset{f=1}{\sum }}\overset{V_{f}}{\underset{v=1}{\sum }}Q_{\mathrm{vf}}^{S}\delta_{\mathrm{ivf}}$$

where, $Q_{\mathrm{vf}}^{S}$ is the areal quantity in the source vertex v, $v\;\in \;\left\{1,\ldots ,V_{f}\right\}$ lying on the target face f, f∈{1,…,F}, F the number of faces that meet at the target vertex i, and $\delta_{\mathrm{ivf}}$ the barycentric coordinate of v, lying on face f, and in relation to the target vertex i. The key difference between this method and the classical barycentric interpolation is that, in the latter, the coordinates of the target vertex in relation to their containing source face are used to weight the quantities, in a process that is not mass conservative. Here barycentric coordinates of the source vertex in relation to their containing target face are used; the quantities are split proportionately, and redistributed across target vertices.

#### Pycnophylactic

The ideal interpolation method should conserve the areal quantities globally, regionally and locally, that is, the method has to be pycnophylactic. This is accomplished by assigning, to each face in the target sphere, the areal quantity of all overlapping faces from the source sphere, weighted by the fraction of overlap between them (Markoff and Shapiro 1973; Winkler et al. 2012). The pycnophylactic method operates directly on the faces, not on vertices, and the area (or any other areal quantity) is transferred from source to target surface via weighting by the overlapping area between any pairs of faces. The interpolated areal quantity, $Q_{i}^{T}$, of a face i in the target surface, that overlaps with F faces from the source surface, is given by the following equation:

$$Q_{i}^{T}=\overset{F}{\underset{f=1}{\sum }}\frac{A_{f}^{O}}{A_{f}^{S}}Q_{f}^{S}$$

where $A_{f}^{S}$ is the area of the f-th overlapping face from the source sphere, which contains a quantity $Q_{f}^{S}$ of some areal measurement (such as the surface area measured in native space), and $A_{f}^{O}$ is the overlapping area with face i.

### Smoothing

For the comparison of the areal interpolation methods and for the volume methods, smoothing was applied at 2 levels: no smoothing, and smoothing with a Gaussian kernel with full width at half maximum (FWHM) of 10 mm, chosen so as to preserve the effect of the different resolutions being investigated. For the comparison between VLBW and controls, 30 mm was used, as in Rimol et al. (2016). Before smoothing, correction for unequal face sizes (Winkler et al. 2012) was applied for all interpolation methods.

### Statistical Analysis

The statistical comparison between VLBW and controls was performed using PALM—Permutation Analysis of Linear Models (Winkler, Webster et al. 2016). The number of permutations was set to 1000, followed by approximation of the tail of the distribution by a generalized Pareto distribution (GPD; Winkler, Ridgway, et al. 2016). Familywise error rate correction (FWER) was done considering both hemispheres and both test directions for the null hypothesis of no difference between the 2 groups. Analyses were performed separately for cortical thickness, area, and volume (both methods), and also using NPC with Fisher’s combination of *P*-values (Fisher 1932) for the joint analysis of thickness and area; Supplementary Material S5 shows an overview of how these analyses are related.

## Results

### Preservation of Areal Quantities

**Figure 3.**
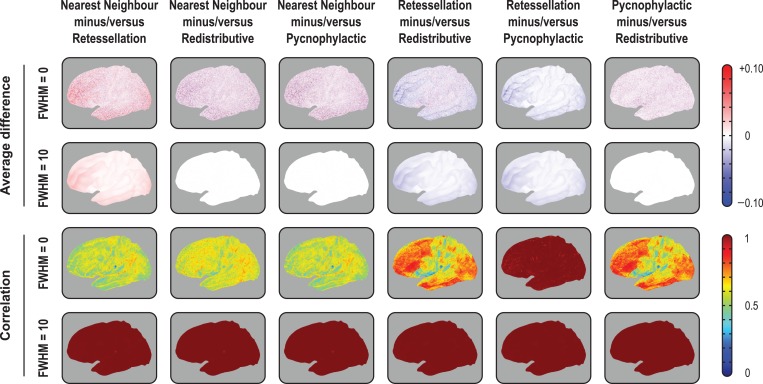
Pairwise average differences (in mm^2^) and correlations between the 4 interpolation methods, using the IC7 as target, with or without smoothing with a Gaussian kernel of FWHM = 10 mm, projected to the average white surface. Although the 4 methods differ, with some leading to substantial, undesirable losses and gains in surface area, and the introduction of noise manifested by lower correlations, the average variation was zero for nearest neighbor, redistributive and pycnophylactic. The retessellation method led to substantial losses of area that could not be recovered or compensated by blurring. Although this method showed excellent correlation with pycnophylactic, quantitative results after interpolation are biased downwards. For the medial views, for the right hemisphere, for IC3 and IC5, and for projections to the pial and inflated surfaces, consult the Supplementary Material.

All methods preserved generally well the global amount of surface area, and therefore, of other areal quantities, at the highest resolution of the common grid (IC7). At lower resolutions, massive amounts of area were lost with the retessellation method: about 40% on average for IC3 (lowest resolution, with 642 vertices and 1280 faces) and 9% for IC5 (intermediate resolution, with 10 242 vertices and 20 480 faces), although only 1% for IC7 (163 842 vertices and 327 680 faces). Areal losses, when present, tended to be uniformly distributed across the cortex (Fig. 3, upper panels), with no trends affecting particular regions and, except for retessellation, could be substantially alleviated by smoothing. With the latter method, areal losses accumulated throughout the cortex, and the global cortical area, if computed after interpolation, became substantially reduced (biased downwards), even at the highest resolution of the common grid. An extended set of results that demonstrate these findings is shown in Supplementary Material S2.

### Differences Between Interpolation Methods

While there were no spatial trends in terms of areal gains or losses, the inexactness of the nonpycnophylactic interpolation methods introduced noise that substantially reduced their correlations when assessed between subjects (Fig. 3, lower panels). The only exception was between the retessellation and the pycnophylactic methods, which had near perfect correlation even without any smoothing. Smoothing increased the correlation between all methods to near unity throughout the cortex (Supplementary Material S2a). At the subject level, the spatial correlation between the nearest neighbor and the pycnophylactic was only about 0.60 without smoothing, although it approached unity when the subjects were averaged (Supplementary Material S2b). Smoothing lead to a dramatic improvement in agreement between the methods, causing nearest neighbor to be nearly indistinguishable from the pycnophylactic method. The redistributive method performed in a similar manner, although with a higher correlation without smoothing, that is, about 0.75 (Supplementary Material S2b).

### Cortical Volume Measurements

**Figure 4.**
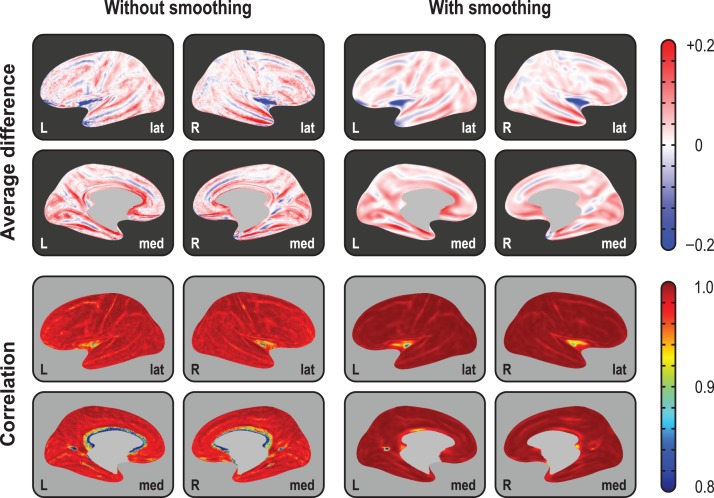
Average difference (in mm^3^) between the 2 methods of assessing volume and their correlation (across subjects), using the highest resolution (IC7) as the interpolation target, projected to the average inflated surface. As predicated from Figure 1, differences are larger in the crowns of gyri and in the depths of sulci, with gains/losses in volume in these locations following opposite patterns. Although the correlations tend to be generally high, and increase with smoothing, they are lower in regions of higher interindividual morphological variability, such as at the anterior end of the cuneus, and in the insular cortex. For IC3 and IC5, and for projections to the white and pial surfaces, consult the Supplementary Material.

At the local scale, differences between the product and the analytic methods of volume estimation were as high as 20% in some regions, an amount that could not be alleviated by smoothing or by changes in resolution. As predicted by Figure 1, differences were larger in the crowns of gyri and depths of sulci, in either case with the reverse polarity (Fig. 4, upper panels). The vertexwise correlation between the methods across subjects, however, was in general very high, approaching unity throughout the whole cortex, with or without smoothing, and at different resolutions. In regions of higher sulcal variability, however, the correlations were not as high, sometimes as low as 0.80, such as in the insular cortex and at the confluence of parieto-occipital and calcarine sulci, between the lingual and the isthmus of the cingulate gyrus (Fig. 4, lower panels). At least in the case of the insula, this effect may be partly attributed to a misplacement of the white surface in the region lateral to the claustrum (Glasser et al. 2016). Supplementary Material S3 includes additional results that support these findings.

### Global Measurements and Their Variability

Average global cortical area, thickness, and volume (using both methods) across subjects in the sample are shown in Table 2. Cortical volumes assessed with the multiplicative method were significantly higher (p<0.0001) than using the analytic method. Variability for area was higher than for thickness, and even higher for volume: the average coefficient of variation across subjects (100σ/μ) was, respectively, 9.9%, 3.2%, and 10.5%, after adjusting for group, age, and sex, with the parietal region (bilateral) being the most variable for all measurements. The corresponding spatial maps, as well as correlation and Bland–Altman plots, are shown in Supplementary Material S4.

**Table 2:** Average ± standard deviation of area (in mm^2^), thickness (in mm) and volume (in mm^3^) across control subjects. Volumes were assessed using the multiplicative (*m*) and analytic (*a*) methods; their difference is also shown

Measure	Left hemisphere	Right hemisphere	Both hemispheres
Area	100877.7 ± 7868.3	101725.3 ± 8101.4	202603.0 ± 15944.4
Thickness	2.5556 ± 0.0896	2.5436 ± 0.0880	2.5495 ± 0.0876
Volume^(*m*)^	257781.8 ± 21973.5	258751.1 ± 22694.0	516533.0 ± 44562.1
Volume^(*a*)^	254053.8 ± 21740.6	255181.4 ± 22441.4	509235.1 ± 44080.0
Difference^(*m−a*)^	3728.0 ± 581.6	3569.8 ± 624.1	7297.8 ± 1108.1

### Differences Between VLBW and Controls

**Figure 5.**
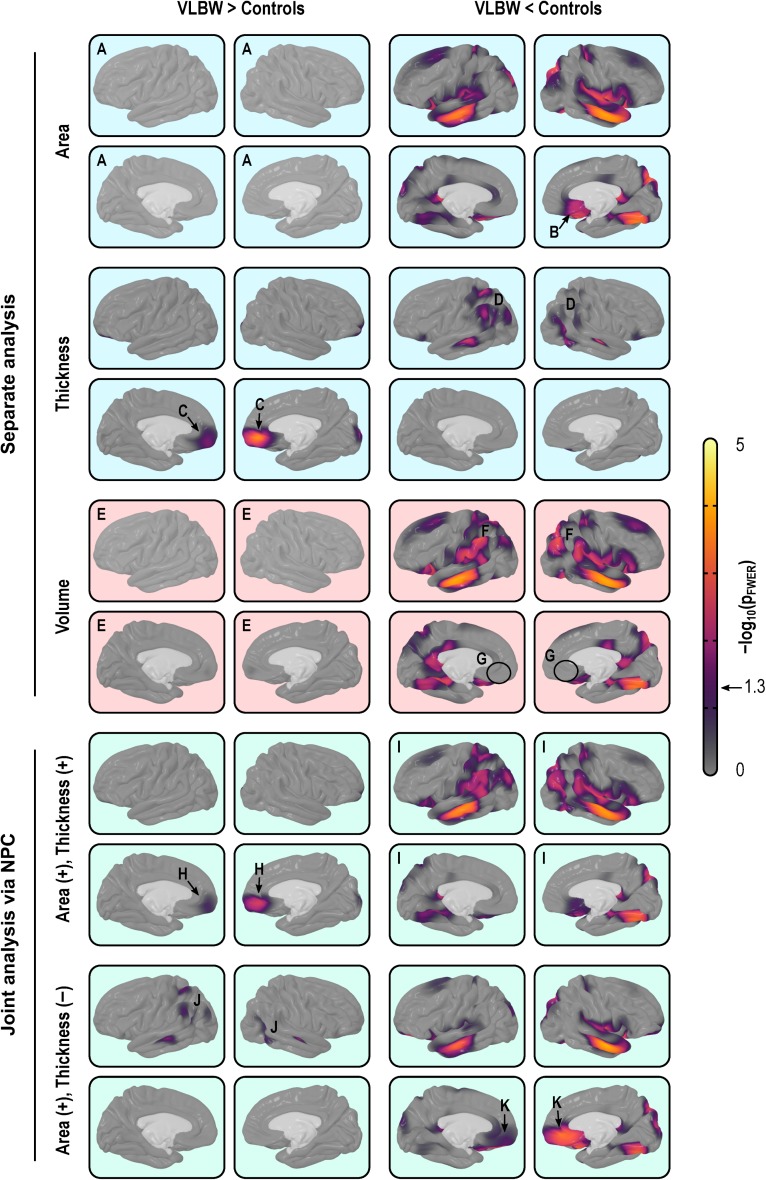
Separate (light blue background) and joint (green) analysis of cortical area and thickness, as well as volume (red), using the IC7 resolution and smoothing with FWHM = 30 mm. Analysis of area indicates no increases in the VLBW group anywhere in the cortex (*A*), and reductions in, among other regions, the subgenual region of the cingulate cortex (*B*). Analysis of thickness indicates that VLBW subjects have thicker cortex in the medial orbitofrontal cortex (*C*) and in the right medial occipital cortex, as well as diffuse bilateral thinning in parietal and middle temporal regions (*D*). Analysis of volume alone broadly mimics analysis of area, with no evidence of increased volume in VLBW subjects (*E*), although in some maps there seems to be a partial superimposition of the effects seen separately for area and thickness, with signs of bilaterally decreased volume throughout the parietal lobe (*F*) but, contrary to the analysis of area, no signs of reduction in the subgenual cortex (*G*). Jointly analyzing area and thickness gives equal weight to both measurements, and allows directional effects to be inferred. Contrary to the case for volume, it is possible to know that there is an increase in the amount of cortical tissue in VLBW subjects in the medial orbitofrontal cortex (*H*) when compared with controls, and a bilateral decrease throughout most of the parietal cortex, stronger in the middle temporal and fusiform gyri, in both hemispheres (*I*). Moreover, the joint analysis allows search for effects that can negate each other, such as in this case weaker effects in the parietal region (*J*), that partially overlap in space with those shown in (*I*). Finally, strong effects in the middle orbitofrontal, which were missed with a simple volume analysis (*G*), become clearly visible (*K*).

Analyzing cortical thickness and area separately, the comparisons between VLBW subjects and term-born controls suggested a distinct pattern of differences. Surface area maps showed a significant bilateral reduction in the middle temporal gyrus, the superior banks of the lateral sulcus, and the occipitotemporal lateral (fusiform) gyrus, as well as a diffuse bilateral pattern of areal losses affecting the superior frontal gyrus, posterior parietal cortex and, in the right hemisphere, the subgenual area of the cingulate cortex. Cortical thickness maps showed a diffuse bilateral thinning in the parietal lobes, left middle temporal gyrus, right superior temporal sulcus, while showing bilateral thickening of the medial orbitofrontal cortex and the right medial occipital cortex of the VLBW subjects compared with controls (Fig. 5, upper panels, light blue background). Maps of cortical volume differences largely mimicked the surface area results, albeit with a few differences: diffuse signs of volume reduction in the parietal lobes, ascribable to cortical thinning and, contrary to the analysis of area and thickness, no effects found in the medial–orbitofrontal or in the subgenual region of the cingulate gyrus (Fig. 5, middle panels, light red background).

### Joint Analysis via NPC

NPC of thickness and area provided information about patterns of group differences not visible in cortical volume analyses (Fig. 5, lower panels, light green background). In the present data, the joint analysis suggested a decrease in the amount of tissue in VLBW subjects in the medial orbitofrontal cortex, which was not visible in the volume analysis, as well as a bilateral decrease throughout most of the parietal cortex, and in the middle temporal and fusiform gyri. In addition, NPC showed simultaneous bilateral decrease in surface area and increase in thickness in the medial orbitofrontal gyrus, none of which was observed using simple volume measurements. Additional maps are shown in Supplementary Material S5.

## Discussion

### Interpolation of Areal Quantities

The different interpolation methods did not perform similarly in all settings. Nearest neighbor and redistributive required smoothing of at least FWHM = 10 mm, as used here, in order to become comparable to, and interchangeable with, the pycnophylactic methods. However, since data is usually smoothed in neuroimaging studies in order to improve the matching of homologies and to improve the signal-to-noise ratio, this is not a significant limitation. Retessellation, particularly at lower resolutions, lead to substantial areal losses that could not be recovered even with smoothing. Moreover, the vertices of the retessellated surfaces are not guaranteed to lie at the tissue boundaries they represent, introducing uncertainties to the obtained measurements. Regarding speed, although the various implementations run in linear time, the pycnophylactic method has to perform a larger number of computations that may not pay off when compared with nearest neighbor, provided that smoothing is used.

### Volumes Improved, Yet Problematic

The large absolute difference between the product and the analytic method for cortical volume indicates that if interest lies in the actual values (for instance, for predictive models), the analytic method is to be preferred. The high correlation across subjects, however, suggests that, for group comparisons and related analyses, both methods generally lead to similar results, except in a few regions of higher morphological interindividual variability. However, even for group comparisons, cortical volume is a poor choice of trait of interest. Even though volume encapsulates information from both area and thickness, research has suggested that the proportion in which the variabilities of these 2 measurements coalesce varies spatially across the cortical mantle (Winkler et al. 2010; Storsve et al. 2014). Moreover, previous literature suggests that most of the between-subject variability in cortical volume, including that measured using VBM, can be explained by the variability of surface area (Voets et al. 2008; Lenroot et al. 2009; Winkler et al. 2010; Rimol et al. 2012), whereas most of the within-subject variability can be explained by variability of cortical thickness, at least during adult life (Storsve et al. 2014), thus rendering volume a largely redundant metric. In effect, the continuous cortical maps in Figure 5, resulting from a between-subject analysis, confirm that the results for cortical volume largely mirror the results for cortical surface area.

### Joint Analyses via NPC

Such problems with cortical volume can be eschewed through the use of a joint statistical analysis of area and thickness. The NPC methodology gives equal (or otherwise predefined) weights for thickness and area, which therefore no longer have their variability mixed in unknown and variable proportions across the cortical mantle. Various combining functions can be considered, and the well-known Fisher method of combination of *P*-values is a simple and computationally efficient choice. By using 2 distinct metrics in a single test, power is increased (Fisher 1932; Pesarin and Salmaso 2010a; Winkler, Webster, et al. 2016), allowing detection of effects that otherwise may remain unseen when analyzing volume, or when thickness and area are analyzed separately. NPC can be particularly useful for the investigation of processes affecting cortical area and thickness simultaneously, even if in opposite directions or at different rates (both phenomena that have been recently reported, e.g., Hogstrom et al. 2013; Storsve et al. 2014), and can effectively replace volume as the measurement of interest in these cases, with various benefits and essentially none of the shortcomings. It constitutes a general method that can be applied to any number of partial tests, each relating to hypotheses on data that may be of a different nature, obtained using different measurement units, and related to each other arbitrarily.

Moreover, NPC allows testing directional hypotheses (by reversing the signs of partial tests), hypotheses with concordant directions (taking the extremum of both after multiple testing correction), and two-tailed hypotheses (with two-tailed partial tests). Power increases consistently with the introduction of more partial tests when there is a true effect, while the error rate is strictly controlled. This is in contrast to classical multivariate tests based on regression, such as MANOVA or MANCOVA, that do not provide information on directionality of the effects, and lose power as the number of partial tests increase past a certain optimal point. Usage of NPC is not constrained to the replacement of cortical volume, and the method can be considered for analyses involving other cortical indices, including myelination (Glasser and Van Essen 2011; Sereno et al. 2013) and folding and gyrification metrics (Mangin et al. 2004; Schaer et al. 2008; Toro et al. 2008) that can interact in distinct and complex ways (Tallinen et al. 2014, 2016), among others. Due to its nonparametric nature, a joint analysis offers an elegant solution to the problem of multiple comparisons, both across locations on the cortical surface (vertices), and across measurements; it also offers increased power over separate analyses.

### Permutation Inference

NPC is based on permutations in each of the partial tests but does not preclude separate analyses of thickness and area, and can accommodate partial tests combining positive and negative directions. Using permutation tests with synchronized shuffling, it is trivial to correct for the multiplicity of tests while taking their nonindependence into account. Permutation tests provide exact inference based on minimal assumptions, while allowing multiple testing correction with strong control over the error rate. Even though these tests still have certain requirements, such as the data being exchangeable, various types of structured dependency can be accommodated by means of restricted permutation strategies. Finally, permutation tests do not depend on distributional assumptions, which is an advantage when analyzing surface area, since area at the local level shows positive skewness and is better characterized as log-normal (Winkler et al. 2012).

### Area and Thickness of VLBW Subjects

The sample used for this analysis is particularly suitable as neurodevelopmental brain disorders associated with preterm birth are known to have a divergent effect on cortical area and cortical thickness, including both cortical thinning and thickening (Rimol et al. 2016), hence a joint analysis being potentially more informative in lieu of simple cortical volume. Here, the reduced cortical surface area observed in VLBW subjects compared with controls replicates previous findings from the same cohort (Skranes et al. 2013), and is consistent with findings from a younger cohort of VLBW subjects (Sølsnes et al. 2015) and teenagers born with extremely low birth weight (≤1000 g) (Grunewaldt et al. 2014). The combined evidence from these studies suggests that surface area reductions in the preterm brain are present from early childhood and remain until adulthood (Rimol et al. 2016), and various mechanisms have been proposed (Volpe 2009, 2011; Eikenes et al. 2011; Hagberg et al. 2015). Likewise, explanations for why the cortex is thinner in some regions and thicker in others, in VLBW subjects, have been proposed (Marín-Padilla 1997; Bjuland et al. 2013; Grunewaldt et al. 2014). The combination of thickening and reduced area in medial orbitofrontal cortex has been observed in multiple cohorts and, in light of previously proposed mechanisms, these changes could be related to prenatal factors, such as fetal growth restriction, or to postnatal exposure to extrauterine environmental stressors (Sølsnes et al. 2015; Rimol et al. 2016). Regardless of underlying pathological factors, the morphological indices appear to be robust markers of perinatal brain injury and maldevelopment (Raznahan et al. 2011; Skranes et al. 2013; Rimol et al. 2016).

### Limitations

As NPC is a permutation test, the assumption of exchangeability must hold, which can be a limitation when certain types dependencies between observations exist. The method can be computationally intensive, particularly for datasets that are large or have high resolution. Both problems can be addressed, at least in particular cases: structured dependencies (such as when studying twins) can be accommodated by imposing restrictions on which permutations can be performed (Winkler et al. 2015), whereas accelerations can be accomplished using various approximate or exact methods (Winkler, Ridgway, et al. 2016); the latter were used in this particular analysis. Regarding power, the present VLBW sample is medium-sized and it is possible that real group effects were not detected, including volume differences. However, to the extent that cortical thickness and surface area go in opposite directions, failure to detect group differences in cortical volume are unlikely to be related to power issues with the volume analysis.

## Conclusion

We studied the 4 extant interpolation methods for the assessment of cortical area, and observed that the nearest neighbor interpolation, followed by a Jacobian correction and smoothing, is virtually indistinguishable from the pycnophylactic method, while offering reduced computational costs. This leads us to recommend, for practical purposes, the nearest neighbor method, with smoothing, when investigating cortical surface area. In addition, we demonstrated that the NPC of cortical thickness and area can be more informative than a simple analysis of cortical volume, even when the latter is assessed using the improved, analytic method that does not over- or under-represent tissue according to the cortical convolutions.

## Supplementary Material

Supplementary Data
