# Supplementary material for: Joint Analysis of Cortical Area and Thickness as a Replacement for the Analysis of the Volume of the Cerebral Cortex
Source: Cereb Cortex. 2017 Nov 28;28(2):738–49. doi: 10.1093/cercor/bhx308 (PMC5972607; doi:10.1093/cercor/bhx308)
Supplement: Supplementary Data [file bhx308_supplement_data.zip › bhx308manuscript_smallfixes.docx]

# Supplementary Material

Detailed results have been organised in a set of browsable pages and packaged into a 7.8 gb file that constitutes the Supplementary Material. It is deposited for long term preservation and public access at the Dryad Digital Repository, under the Digital Object Identifier (doi): [10.5061/dryad.h1v85](http://dx.doi.org/10.5061/dryad.h1v85). The results above make ample reference to this material, and its inspection is encouraged. The Supplementary Material also includes high resolution and complementary views of all figures shown in the main text. A mirrored copy that does not require download, though not guaranteed for permanent preservation, can be found at <http://bit.ly/2x9F96b>.
